# Thermal-inert and ohmic-contact interface for high performance half-Heusler based thermoelectric generator

**DOI:** 10.1038/s41467-022-35290-6

**Published:** 2022-12-14

**Authors:** Ruiheng Liu, Yunfei Xing, Jincheng Liao, Xugui Xia, Chao Wang, Chenxi Zhu, Fangfang Xu, Zhi-Gang Chen, Lidong Chen, Jian Huang, Shengqiang Bai

**Affiliations:** 1 State Key Laboratory of High Performance Ceramics and Superfine Microstructure, Shanghai Institute of Ceramics, Chinese Academy of Sciences, Shanghai, 200050 China; 2 Center of Materials Science and Optoelectronics Engineering, University of Chinese Academy of Sciences, Beijing, 100049 China; 3 Shenzhen Institute of Advanced Electronic Materials, Shenzhen Institutes of Advanced Technology, Chinese Academy of Sciences, Shenzhen, 518055 China; 4 School of Chemistry and Physics, Queensland University of Technology, Brisbane, QLD 4001 Australia

**Keywords:** Thermoelectrics, Thermoelectric devices and materials

## Abstract

Unsatisfied electrode bonding in half-Heusler devices renders thermal damage and large efficiency loss, which limits their practical service at high temperatures. Here, we develop a thermodynamic strategy to screen barrier layer elements. Theoretically, we found that the interface between VIIB elements and half-Heuslers possesses near-zero interfacial reaction energy and large atomic diffusion barrier. Experimentally, such an interphase proves to be the atomic direct bonding and has high thermal stability at 1073 K, leading to ideal ohmic contact. Such thermally inert and ohmic contact interface enable modules stably to work at elevated temperature up to 1100 K, which releases the peak performance of half-Heuslers and in turn boosts the energy conversion efficiencies to the records of 11.1% and 13.3% for half-Heusler single-stage and half-Heusler/Bi_2_Te_3_ segmented modules. This design strategy provides a feasible solution for the high-temperature half-Heusler generators and gives enlightenment for other package interconnection design of electronic devices.

## Abstract (web summary)

Unsatisfied electrode bonding in half-Heusler devices limits their practical service at high temperatures. Here, the authors develop a direct bonding interface to ideal ohmic contact and thermally inert and ohmic contact, leading to high deficiency.

## Introduction

To achieve global carbon emission target, low carbon technology has attracted more and more attention in both scientific and industrial community. Thermoelectric (TE) technology provides an environmental-friendly solution to the recovery of low-grade thermal energy by converting it directly into useful electricity based on Seebeck effect without moving parts or emissions. To promote this technique to real industrial applications, the primary task is to enhance the energy conversion efficiency (*η*) of TE devices, given by ref. 1$\eta=\left(\frac{{T}_{{{{{\rm{h}}}}}}-{T}_{{{{{\rm{c}}}}}}}{{T}_{{{{{\rm{h}}}}}}}\right)\left[\frac{\sqrt{1+Z\bar{T}}-1}{\sqrt{1+Z\bar{T}}+\left(\frac{{T}_{{{{{\rm{c}}}}}}}{{T}_{{{{{\rm{h}}}}}}}\right)}\right]$, where *Z* is the figure of merit of TE materials, ${T}_{{{{{{\rm{h}}}}}}}$, ${T}_{{{{{{\rm{c}}}}}}}$, and $\,\bar{T}$ are the hot-side, cold-side and average temperatures of TE devices, respectively. For achieving high *η*, it is essential to use TE materials with large *ZT* and high-temperature heat source applying for large temperature drops across the device.

In the past decades, continuous efforts have been made to enhance the performance of TE materials, and the dimensionless figure of merit (*zT*) have been greatly promoted over 1.5 and sporadically over 2.0 in lots of novel TE materials such as high-entropy Pb(Se/Te)^2,3^, GeTe^4,5^, half-Heusler^6^ and Mg_3_(Sb/Bi)_2_^7,8^. Among them, half-Hesuler (HH) materials arouse great interest as the exhibited high *zT* values and excellent thermal stability in the high-temperature range over traditional SiGe alloys^4,9–11^. Several typical HH compounds, such as *M*NiSn, *M*CoSb (*M* = Ti, Zr and Hf), and NbFeSb compounds, show very high melting points above 1700 K^6^, and are expected to work stably at high temperatures as 1300 K for long-term. High melting points and high *zT* values enable HH materials significant advantage to realize high *η* under large temperature difference, which potentially promote the application fields, such as radioisotope thermoelectric generators (RTGs) as the power supply in deep space exploration. Nevertheless, only “high-*zT*” is not sufficient to obtain a practical device with high *η*. Another bottleneck is the bonding technology to ensure low energy loss, practical thermal/mechanical stability especially for the hot-side electrode.

Owing to higher thermal and electrical conductivities of HH family, the energy loss caused by the interfacial resistance in HH devices is more severe than those of other TE materials, such as skutterudites^12,13^, and PbTe^1^. Metal elements, such as Ag, Cu, Ti and AgCuSn-based solders, have been reported to interconnect HH material with electrodes^14–18^, and the interface resistivity has been found to increase up to 30 μΩ cm^2^ after aging at 800~1000 K^14,15^. To reduce the interfacial resistivity and to improve the long-term interfacial durability, a general solution is to introduce a metallic barrier, which can suppress chemical reaction and elements diffusion between electrodes and TE matrix^19–21^. Empirically, refractory metals or polynary intermetallic compounds are usually adopted to inhibit the growth of the reaction layer at the interface, such as Nb barrier for filled-CoSb_3_^21^, and Mg_2_Cu barrier for Mg_3_(Sb/Bi)_2_^22^. Nevertheless, conservative measure is still required in practice to suppress interfacial diffusion and/or reaction during service by lowering operation temperatures^23,24^, which is unavoidable to sacrifice certain energy efficiency. Therefore, developing a bonding technology with an ohmic contact and without any interfacial reaction is an ideal goal in the fabrication of TE device. To achieve this goal, the structural features for the ideal interface between TE material and electrode should be involved: thermodynamically inert, direct interatomic bonding, less or no electron scattering across the interface^7,12^. In the previous study^13^, the interfacial reaction energy (*E*_IR_) and activation energy barrier of migration (*E*_Mig_) in the interfacial reaction layers of active atoms (i.e. Sb in skutterudites) were proposed as the criterions for screening the suitable barriers in skutterudite-based device. A reasonably low *E*_IR_ can degenerate the interfacial reaction layer and in turn achieve robust bonding strength of electrode, while a high *E*_Mig_ benefits to realizing low growth rate of interfacial reaction layers and thus suppressing the increase of interfacial resistivity during long-term service.

**Fig. 1:**
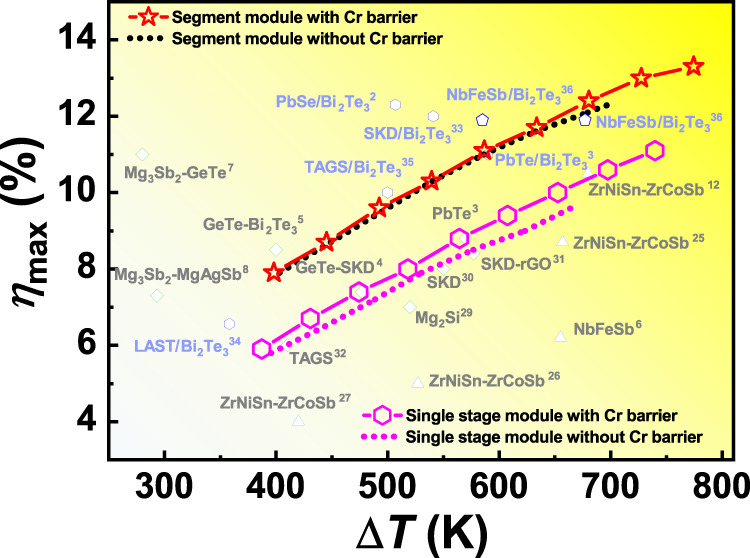
Comparison of module efficiencies. Maximum energy conversion efficiency (*η*_max_) versus temperature difference (*ΔT*) for the HH-based single-stage and segmented modules. Some representative achievements in other novel TE modules are also illustrated for comparison, including the single-stage models of half-Heusler^6,12,29,37–39^, Mg_2_Si^40^, skutterudite (SKD)^41,42^, Pb–Te based^3^, Ge–Te based^4,5,43^, Mg_3_Sb_2_^7^^,^^8^, and various segmented modules of PbSe/Bi_2_Te_3_^2^, PbTe/Bi_2_Te_3_^3^, SKD/Bi_2_Te_3_^34^, LAST/Bi_2_Te_3_^44^, TAGS/Bi_2_Te_3_^45^ and HH/Bi_2_Te_3_^46^. The published data of these modules are summarized in Table S4.

In this work, we systematically investigated the thermodynamics and electrical transportation of various interfaces constructed between candidate barriers (Cr, Mo, W, Cu, Ag, and Au) and typical HH compounds *M*NiSn and *M*CoSb (*M* = Ti, Zr, and Hf). Near-zero *E*_IR_ and high *E*_Mig_ guide the ideal electrode interface microstructure in *M*NiSn and *M*CoSb (*M* = Ti, Zr and Hf) TE devices. The achieved ideal ohmic contact between Cr (Mo) and p-type *M*CoSb induces the disordered boundary between Cr and n-type *M*NiSn to suppress the Schottky barrier. A clean atomic bonding interface and extremely low interfacial resistivities of less than 1 μΩ cm^2^ are maintained even after 30 days aging at 1073 K. By using Cr as the electrode barrier and choosing Zr_0.5_Hf_0.5_NiSn_0.99_Sb_0.01_ and Zr_0.5_Hf_0.5_CoSb_0.8_Sn_0.2_ as n- and p-legs, we fabricate an 8-pair single-stage HH module and an 8-pair segmented HH/Bi_2_Te_3_ module. These modules show stable output performance at an elevated temperature of 1073 K, which covers the peak *zT* corresponded temperatures of HH compounds and is more than 100 K higher than the previously reported modules without Cr barriers. Moreover, both our single-stage and segmented HH modules achieve the record *η* of 11.1% and 13.3% at the temperature difference of 740 K and 774 K, respectively, which is 7~18% higher than the same type of modules without Cr barrier layer (as shown in Fig. 1). This study proves that chemically inert barriers can achieve ideal electrode interface for high-temperature TE devices, which greatly promotes the service temperature and output performance of HH-based modules. This study also gives enlightenment for other high-temperature TE materials and other fields such as package interconnection design of electronic devices.

## Results and discussion

**Fig. 2:**
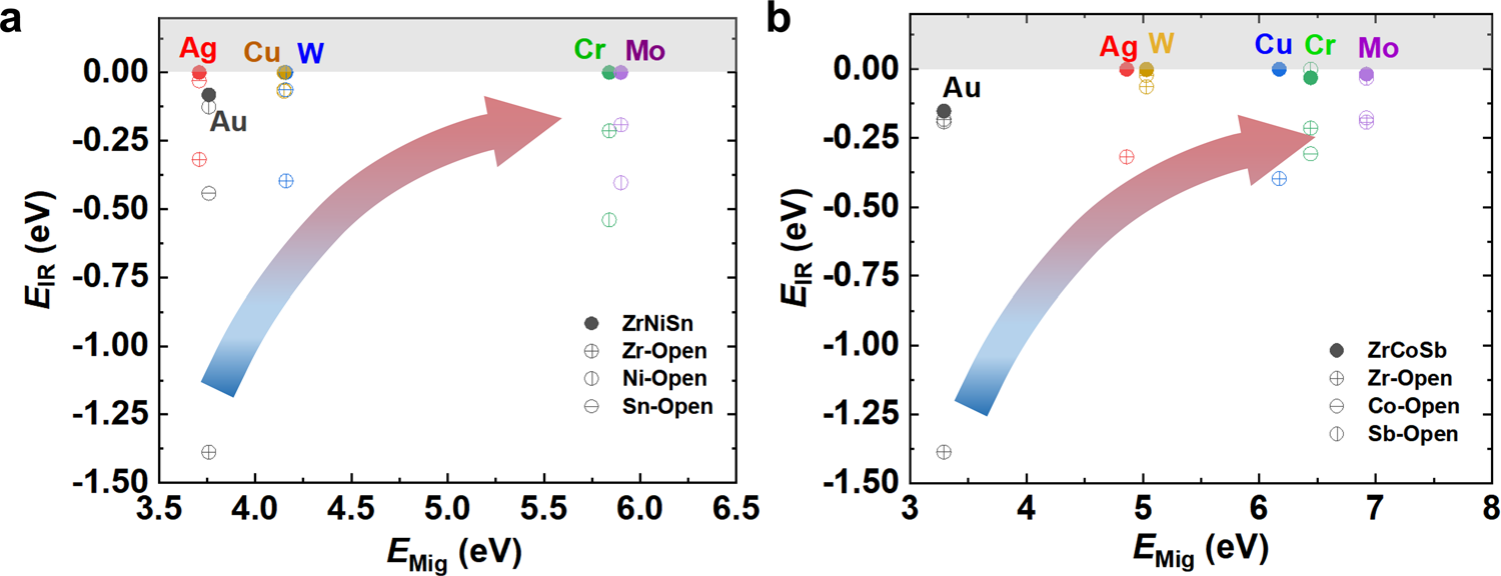
Thermodynamic strategy to screen barrier layer elements. The interfacial reaction energy (*E*_IR_) and activation energy barrier (*E*_Mig_) of IB group (Cu, Ag and Au) and VIB group (Cr, Mo, and W) elements in **a** ZrNiSn and **b** ZrCoSb. The arrows suggest the screening strategy for ideal barrier materials.

The interfacial reaction energies (*E*_IR_) for both n-type ZrNiSn and p-type ZrCoSb HH systems using various metals as contacting layer are shown in Fig. 2. In order to design the ideal electrode interface with an ohmic contact, we choose IB group (Cu, Ag and Au) and VIB group (Cr, Mo, and W) elements as the candidates, since these two groups’ elements showed inertia as dopants in ZrNiSn- and ZrCoSb-based HHs while possessing relative high melting point above 1200 K. The common interface evolution contains two consecutive and coupling processes: chemical reaction and the diffusion^25^, governed by thermodynamic and kinetic factors^13^. The interfacial chemical reactions between ZrNiSn(ZrCoSb) and *d*-metal candidates were firstly investigated following the methodology proposed by Ceder et al.^26^. As shown in Fig. 2, if the component elements of ZrNiSn & ZrCoSb were considered as in closed system, the selected *d*-metals except Au could hardly react with ZrNiSn & ZrCoSb, since almost all of the calculated interfacial reaction energies (*E*_IR_) are approaching to zero. We further examined the *E*_IR_ in different open conditions and found that, for IB group metals M^IB^ (Cu, Ag, and Au), Zr-open condition could greatly increase the *E*_IR_ of both M^IB^/ZrNiSn and M^IB^/ZrCoSb joints, while for VIB group metals M^VIB^ (Cr, Mo, and W), Co-open or Ni-open could significantly increase the *E*_IR_ of M^VIB^/ZrNiSn and M^VIB^/ZrCoSb joints, respectively. Generally, the more negative value of *E*_IR_ is, the more easily interface reaction would occur, which is not preferred for barrier materials due to the fast growth of the interface. Among all these *d*-metals, Cr maintains the nearly zero *E*_IR_ for all closed and open conditions, showing the most potential as the barrier layer for ZrNiSn and ZrCoSb.

In order to illustrate the nonequilibrium interdiffusion behavior on the interfacial property, we further investigated the possible interdiffusion between all the selected candidate barriers and HH compounds. As shown in Supplementary Table 2, most candidate elements prefer to occupy the tetrahedral vacancy site of HH lattice except Ag and Au. Thus, the migration channels for VIB metals and Cu are considered as from one tetrahedral vacancy site to neighboring unoccupied vacancy site in both ZrNiSn and ZrCoSb systems, while the migration channel for Au in ZrNiSn is considered as from Ni site to neighboring Ni vacancy site, and the migration channels for Ag and Au in ZrCoSb are considered as from Sb site to neighboring Sb vacancy site. The activation energy barrier (*E*_Mig_) of *d*-metals along the corresponding channels can be determined by the CI-NEB method^27^. As shown in Fig. 2, all the *d*-metals exhibit high migration energy barriers above 3.0 eV in both ZrNiSn and ZrCoSb lattices, and Cr and Mo have the highest *E*_Mig_ above 6.0 eV. All these values are much higher than the self-diffusion energy barriers of *d*-metals (see Supplementary Fig. 1), indicating that the diffusion is a one-way migration from the *d*-metal side to HH side. Since the diffusion across the interface is usually much faster than the diffusion in the bulk materials, the diffusion in HH is supposed to control the overall kinetic process^25^. Therefore, screening promising barrier layer(s) for HH compounds can be followed by the criteria: (1) a negative *E*_IR_ but as small as near-zero to ensure reasonable bonding but avoid severe interfacial chemical reaction, and (2) a large *E*_Mig_ enough to restrict the atomic diffusion. Based on these criteria, Mo and Cr are screened out from the considered pure metals as the best candidates of diffusion barrier layer for both p- and n-type HH materials.

**Fig. 3:**
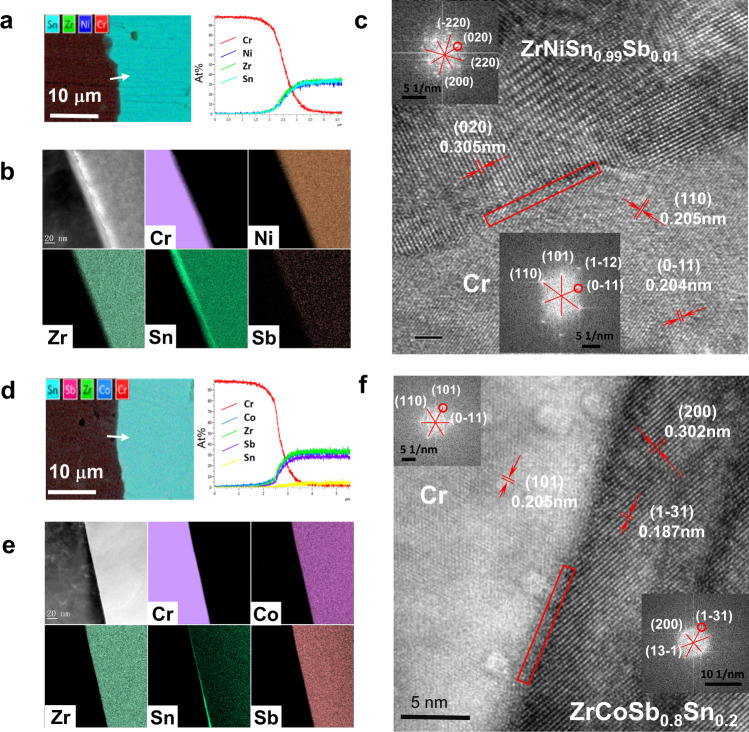
Microstructures of bonding interface. Microstructures of as-prepared Cr/ZrNiSn_0.99_Sb_0.01_ and Cr/ZrCoSb_0.8_Sn_0.2_ joints: **a** Elemental mapping and composition profiles along the arrow-line in SEM. **b** High-angle annular dark-field (HAADF) image and the corresponding EDS elemental mapping. **c** High-resolution TEM to show the interface matching between Cr and ZrNiSn_0.99_Sb_0.01_ phases, insets: Fourier transformation images corresponding to Cr and ZrNiSn_0.99_Sb_0.01_, respectively. **d** Elemental mapping and composition profiles along the arrow line in SEM. **e** High-angle annular dark-field (HAADF) image and the corresponding EDS elemental mapping. **f** High-resolution TEM to show the interface matching between Cr and ZrCoSb_0.8_Sn_0.2_ phases, insets: Fourier transformation images corresponding to Cr and ZrCoSb_0.8_Sn_0.2_, respectively.

According to the above prediction, we fabricated four kinds of joints, i.e. Cr/ZrNiSn_0.99_Sb_0.01_, Mo/ZrNiSn_0.99_Sb_0.01_, Cr/ZrCoSb_0.8_Sn_0.2_, and Mo/ZrCoSb_0.8_Sn_0.2_. All these joints were aged at 1073 K in vacuum for 30 days. As shown in the element mapping images of SEM (Fig. 3a, d and Supplementary Fig. 2 and 3), there are no observable interfacial reaction products in micrometer scale. Furthermore, as shown in Fig. 3b, e, the HADDF images represent sharp and clean interfaces in both Cr/ZrNiSn_0.99_Sb_0.01_ and Cr/ZrCoSb_0.8_Sn_0.2_ joints, and the nanometer-level elemental mapping results further indicate no detectable reaction product except trace of Sn precipitation, which is probably caused by reaction with residue during synthesis. The high-resolution STEM images further confirm the direct atomic bonding, where the interface in the observed scope is constructed by (211)_Cr_ plane and (020)_ZrNiSn_ plane for Cr/ZrNiSn joint as shown in Fig. 3d and Supplementary Fig. 6c. Meanwhile, the lattice mismatch between (110)_Cr_ and (020)_ZrNiSn_ planes at the interface Cr/ZrNiSn (or (110)_Cr_ plane and (131)_ZrCoSb_ plane for Cr/ZrCoSb joint) is quite large. As shown in Fig. 3c and 3f, the lattice space of (110)_Cr_ is 2.04 Å, the plane space of (020)_ZrNiSn_ is 3.05 Å, and the plane space of (131)_ZrCoSb_ is 1.87 Å. The mismatch is about 50% between (110)_Cr_ and (020)_ZrNiSn_ planes, and about 10% between (110)_Cr_ and (131)_ZrCoSb_ planes. The large plane mismatches suggest that it is difficult to form coherent interfaces (usually <5% of plane mismatch) between Cr and p-type or n-type HH compounds^28^. The STEM images in Fig. 3c, f also indicate the disordered arrangement of atoms adjacent to the interfaces, confirming the incoherent or semi-coherent characters of the interfaces between Cr and current HH compounds.

The 3-dimensional atom probe (3D-ATP) investigation was further employed to examine the elements distribution of Cr/ZrNiSn_0.99_Sb_0.01_ joint, and the results are shown in Supplementary Fig. 4. The sharp line scanning result across the interface suggests the absence of intermediate phases. More importantly, the concentration of Cr within ZrNiSn bulk is extremely low, indicating that the diffusion of Cr towards HH side is negligible. At the same time, the concentrations of HH constituent elements in the Cr lattice is also very low, indicating that the Cr-contacting affects hardly the microstructure nor doping level of HH compounds.

**Fig. 4:**
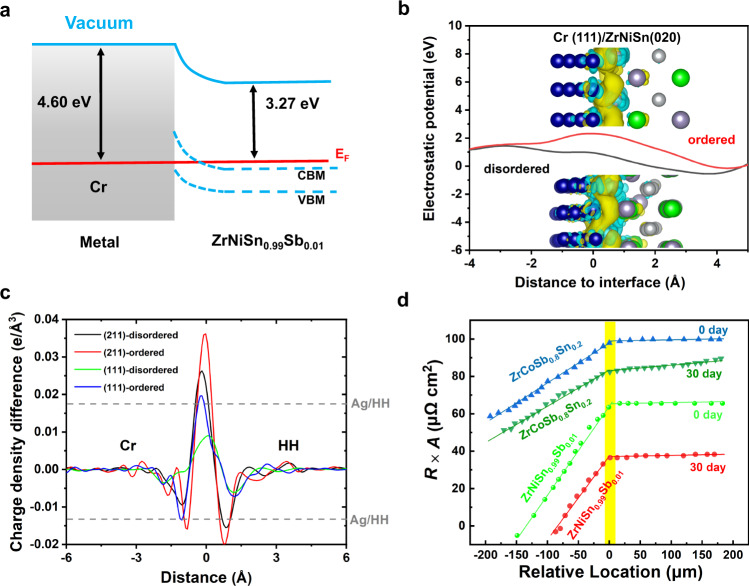
Electrical properties of bonding interface. **a** Schematic band alignment at the interface between Cr and n-type ZrNiSn_0.99_Sb_0.01_, the calculated work functions of Cr and HH are adopted from previous refs. 29, 30. **b** Macroscopic electrostatic potential at the ordered/disordered interfaces between Cr and HH, where the charge density difference around the interface is also shown, plot using a 0.005 e·Å^−3^ isosurface. Blue, gray, silver and green atoms represent Cr, Sn, Ni and Zr, respectively. **c** Planar charge density difference as a function of z-axis distance of the interface for different boundary structures between ZrNiSn and Cr; gray reference lines are corresponding to the peak values of Ag/ZrNiSn^30^; **d** The normalized resistance (*R* × *A*, *A* is the cross section area of the measured joints) scans of Cr/ZrNiSn_0.99_Sb_0.01_ and Cr/ZrCoSb_0.8_Sn_0.2_ joints before and after aged at 1073 K for 30 days.

The results of interfacial resistivity measurement show that the as-fabricated four joints (Cr/ZrNiSn_0.99_Sb_0.01_, Mo/ZrNiSn_0.99_Sb_0.01_, Cr/ZrCoSb_0.8_Sn_0.2_, and Mo/ ZrCoSb_0.8_Sn_0.2_) are all ohmic contact without detectable interfacial resistance, as shown in Fig. 4d, Supplementary Figs. 5 and 6. More excitingly, after aging at 1073 K for 30 days, the interfacial resistance is no change, indicating extreme durability. It is noteworthy that the aging temperature of 1073 K is 100 K higher than the maximum operating temperature of HH module without Cr barriers previously reported^29^. Such marvelous stability of interfacial resistance is attributable to the extreme inertness of interface structure discussed above (Fig. 3 and Supplementary Figs. 2–4). In the resistance scan lines for all as-prepared and aged joints, no resistance drop across the interface can be detected within the voltage test limitation and scan-step precision (~10 μm). The interfacial resistivity, if existing as extra transport barrier, is estimated as low as less than 1 μΩ cm^2^.

The ohmic contact discovered in incoherent interfaces encourages us to investigate the atomic arrangement and its influence on the transport behavior in Cr/HH and Mo/HH interfaces. Firstly, if the atomic interaction at interface is neglected, the classic Schottky-Mott rule can be applied to estimate the band bending of the Cr/HH system by simply comparing the electrode work function and the electron affinity of the HHs^23,30–32^. Based on the estimated work functions and band levels of HHs^41^, the work functions of current HH compounds are evaluated to be ~3.37 eV for ZrNiSn_0.99_Sb_0.01_ and ~3.27 eV for ZrCoSb_0.8_Sn_0.2_, respectively. As shown in Fig. 4a, the schematic band alignments suggest that Cr could form ideal ohmic contact with p-type HH, but form Schottky contacts with n-type HHs with the barrier height ($\varphi$ in Fig. 4a) of ~1.2 eV. However, the Schottky-Mott rule works only in the idea interface without any atomic disorder, while the disordered arrangement of atoms around the interface as observed in Fig. 3 shall significantly pin the Fermi level and thus influence the band alignment. The disordering effect was further verified by the calculated macroscopic electrostatic potential, as shown in Fig. 4b. As can be seen, the analyzed potential from Cr to HH side changes more gently with smaller relevant value at the disordered interface than that at the ordered interface. Both the atomic positions and charge density difference show obvious rearrangement around the interface. We further implemented the ab initio calculations to clarify the electron transportation at such incoherent interface and other possible interfacial configurations (see in Fig. 4, Supplementary Figs. 7–9). Considering the random matching orientations, two interfacial configurations including Cr(211)/ZrNiSn(020) and Cr(111)/ZrNiSn(020) were constructed. Figure 4b shows the charge density differences along the direction perpendicular to interface after full atomic relaxation. Both of the atomic arrangements at the interface show obvious distortion including a number of uniform bonds, and the charge density distribution also present an irregular profile (Supplementary Fig. 7). Figure 4c shows that the larger variation of charge density only occurs within 3 Å at the interface, where band bending is dominated by the interfacial charges. Surprisingly, the disordered interfaces exhibit small peak values than its corresponded ordered one. In other words, the atomic disordering effectively reduces the fluctuation of charge density for the electrons transferring through the interface from Cr side to ZrNiSn side, and therefore weakens the electron scattering. This result is consistent with the previous result in Ag/HH interface, i.e. disordered interface could significantly reduce the Schottky barrier^31^. Meanwhile, the peak value of charge density difference for disordered Cr(211)/ZrNiSn(020) interface is close to that of Ag/HH interface, and the disordered Cr(111)/ZrNiSn(020) interface show only a half value of charge density difference. All these calculation results support the significant role of incoherent interfaces for bending the bands up and rendering almost ohmic contact.

**Fig. 5:**
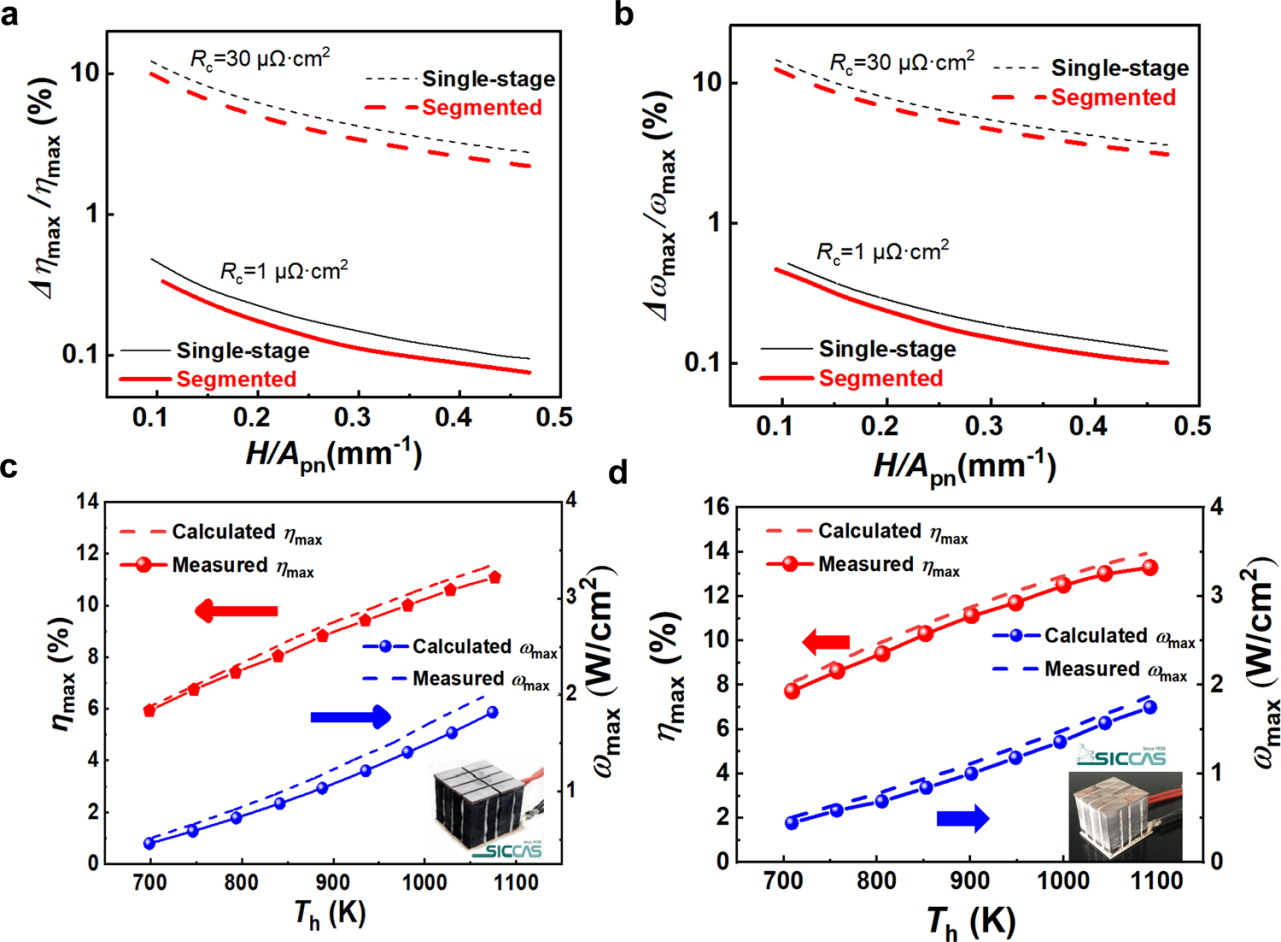
Output performance of module. **a** Calculated loss of maximum conversion efficiency *∆η*_max_/*η*_max_, **b** calculated loss of maximum power density *∆ω*_max_/*ω*_max_ as a function of *H/A*_pn_ and interfacial resistivity (*R*_c_) for different modules. The measured maximum conversion efficiency and power density as a function of *T*_h_ for **c** the HH single-stage module and **d** HH/BT segmented module.

The ultra-low interfacial resistivities play superior role in integrating highly efficient n-ZrNiSn/p-ZrCoSb TE modules. We further used three-dimensional numerical analysis model to evaluate the interfacial effect on maximum conversion efficiencies and power density for both single-stage HH and segmented HH/Bi_2_Te_3_ modules, which were assembled by Zr_0.5_Hf_0.5_NiSn_0.99_Sb_0.01_ and Zr_0.5_Hf_0.5_CoSb_0.8_Sn_0.2_ as n- and p-legs, respectively. The Seebeck coefficient, electrical conductivity and thermal conductivity for Zr_0.5_Hf_0.5_NiSn_0.99_Sb_0.01_, Zr_0.5_Hf_0.5_CoSb_0.8_Sn_0.2_ and commercial Bi_2_Te_3_ materials can be found in our previous work^29^. As shown in Fig. 5a, b, with decreasing the factor *H/A*_pn_ (where *H* and *A*_pn_ is the height and total cross section of TE legs, respectively), the efficiency loss (*∆η*_max_/*η*_max_) and power density loss (*∆ω*_max_/*ω*_max_) caused by interfacial resistivity prominently emerge for both single-stage and segmented modules. When the interfacial resistivity is 30 μΩ cm^2^ (the average value for the interface with Ag–Cu solers)^14,15,29^, the *∆η*_max_/*η*_max_ and *∆ω*_max_/*ω*_max_ is higher than 10% when *H/A*_pn_ = 0.1 mm^−1^. In term of the HH/Cr joints with extremely low interfacial resistivity (~1 μΩ cm^2^), the *∆η*_max_/*η*_max_ and *∆ω*_max_/*ω*_max_ are less than 1%, which exhibits significant advantage for the devices. Meanwhile, the efficiency losses of segmented module are not so sensitive to the interfacial resistance as of single-stage module. This is because the Cr barriers add on the both ends of HH legs in either segmented and single-stage module, and the electrical conductivities of HH materials are higher than that of the Bi_2_Te_3_ in the same service temperature range. The optimized structures of single-stage HH module and HH/Bi_2_Te_3_ segmented module are as shown in Supplementary Fig. 10c, d, where the optimized *A*_p_*/A*_n_ is maintained the same value of 1.6 for both single-stage and segmented modules, while the optimized leg height ratio (*H*_p-HH_*/H*_p-BT_ and *H*_n-HH_*/H*_n-BT_) in segmented module for both p- and n-legs are 6. Thus, the simulated *η*_max_ reaches 11.6% (@ *T*_h_ = 1078 K) for the single-stage HH module, and is 13.9% (@ *T*_h_ = 1090 K) for the HH/Bi_2_Te_3_ segmented module. As shown in Fig. 5c, d, the experimentally measured *η*_max_ of 8-pair single-stage HH module and HH/Bi_2_Te_3_ segmented module reaches the record values of 11.1% and 13.3%, respectively, which are 18% and 7% higher than that of the same modules without Cr barriers.

In this study, the microstructure evolution thermodynamics and electrical transport behavior of interfaces constructed between typical half-Heusler (HH) compounds (ZrNiSn and ZrCoSb) and selected metals (IB group and VIB group) as barrier layer aiming at advancing bonding technology of HH-based TE device. On the basis of the design principle of interface (moderate reaction activity and low atom migration) proposed previously, an advanced design strategy – near-zero interface reaction energy (*E*_IR_) and as large as activation energy barrier of atom migration (*E*_Mig_) – is innovatively proposed for realizing thermally inert direct bonding in HH. According to the first-principles thermodynamic calculation, Cr and Mo are screened out. Different from the well-studied bonding interfaces of such as skutterudites and Bi_2_Te_3_-based TE devices, the interfaces in the fabricated Cr(Mo)/HH joints are of direct atomic connecting without formation of reaction layers keeping inert even after long-term aging at 1073 K. The high-resolution TEM observation reveals the incoherent atomic arrangement at the interface, and the interfacial resistance measurement showing undetectable contacting resistivity. The combination of the incoherent interface benefits to suppress the interfacial electron scattering, and the matching work function between the barrier layer and HH contributes to realizing such ideal ohmic contact. Finally, using Cr as barrier layer and composition-optimized HH materials (n-type ZrNiSn_0.99_Sb_0.01_, p-type ZrCoSb_0.8_Sn_0.2_), the single-stage HH module and HH/Bi_2_Te_3_ segmented module have been fabricated and their conversion efficiencies reach 11.1% and 13.3% at an elevated hot-side temperature closed to 1100 K. The realization of thermally inert and ohmic contacting interfaces in this study provides an effective solution for high-temperature TE power generation with high efficiency and high reliability.

## Methods

### The fabrication and measurement of joints and module

Four kinds of HH materials, i.e. ZrNiSn_0.99_Sb_0.01_, Zr_0.5_Hf_0.5_NiSn_0.99_Sb_0.01_, ZrCoSb_0.8_Sn_0.2_, Zr_0.5_Hf_0.5_CoSb_0.8_Sn_0.2_, were prepared by the self-propagating high-temperature synthesis (SHS) method^33^. For interfacial resistivity investigation, ZrNiSn_0.99_Sb_0.01_ or ZrCoSb_0.8_Sn_0.2_ powder, Cr powder (Alfa Aesar, 99.99%, 25 ± 15 μm) or Mo foil (China New Metal Materials Technology, 99.9%, 100 μm) were loaded into a graphite die of 30 mm inner diameter sequentially and sintered at 850 °C under 60 MPa for 30 min. After sintering process, samples were cut into dices with size of 3 mm × 3 mm × 6 mm, and then were sealed into quartz tubes in vacuum for the aging test. For each aging temperature, there are at least three parallel specimens in one tube. The interfacial microstructure and chemical composition of joints were firstly analyzed in micrometer-level by field emission electron microscopy (FESEM, ZEISS SUPRA 55) and energy dispersive spectroscopy (EDS, OXFORD Aztec X-max80), and the nanometer-level morphology and chemical compositions were investigated with scanning transmission electron microscopy (STEM, Hitachi HF5000) and 3D-atom probe tomography analysis in CAMECA instrument (LEAP 4000X Si). The interfacial resistivity of each sample was tested using a home-built 4-probe platform at least three different locations on the surface, taking the averaged value as the final data.

Both single-stage and segmented modules were assembled by a soldering process. The module employed Zr_0.5_Hf_0.5_NiSn_0.99_Sb_0.01_ as n-type leg, Zr_0.5_Hf_0.5_CoSb_0.8_Sn_0.2_ as p-type leg, and the n- and p-type commercial bismuth tellurides was provided by the Ferrotec Corporation. The legs were diced into the designed dimensions and soldered by a copper plate at the hot end, while the cold end was welded to a direct bonding copper substrate using SAC305 solder. The welding between Bi_2_Te_3_ and Half-Heusler in the segmented module used Sn-based solder. All modules consisted 8-pair of p-n couples with the same envelope area of 20 mm × 20 mm. To reduce the heat loss caused by convection and radiation, glass fibers (HTI1100, Promaglaf) were used to fill the gaps between the legs. The conversion efficiency and output power of each module were tested in a home-built test system under an argon atmosphere^34^.

### Calculation details

We carried out the density functional theory (DFT) calculation using the Vienna Ab initio Simulation Package (VASP)^35^. The Perdew–Burke–Ernzerhof (PBE) functional and projector augmented wave (PAW) pseudopotentials were employed to calculate the total energy. A cutoff energy of 380 eV was fixed throughout all calculations, and a 9 × 9 × 9 k-point mesh was used to sample the Brillouin zone by the Monkhorst method. The numerical thresholds for the convergence of total energy and force on each atom were set to 10^−7^ eV and 0.005 eV/Å, respectively. Lattice and all atoms were fully relaxed by a conjugate-gradient (CG) algorithm. The interfacial chemical reaction between HH and pure d-metal was first considered following the methodology proposed by Ceder et al.^26^. The difference between the close and open conditions is that the reaction energies will be normalized by the number of non-X atoms while the interface system is only open to X atoms. And the energy barriers for atom migration (*E*_Mig_) to neighboring vacancy are determined by CI-NEB method with eight inserted images^36^. The metal/HH interface was built by combining the (020) plane of HH and the (111) or (211) plane of pure metal, where the interface mismatch was controlled less than 5%.

### Finite element simulation

The simulation process was carried out by ANSYS-Workbench. The the temperature-dependent conductivity, Seebeck coefficient and thermal conductivity of HH alloys and commercial Bi_2_Te_3_, and other accessory materials were input to the model. The interfacial resistivities were adopted from our previous research results.

## Supplementary information

Supplementary Information

## Data Availability

All data generated are available from the corresponding author on reasonable request.
