# Supplementary material for: Thermal-inert and ohmic-contact interface for high performance half-Heusler based thermoelectric generator
Source: Nat Commun. 2022 Dec 14;13:7738. doi: 10.1038/s41467-022-35290-6 (PMC9751268; doi:10.1038/s41467-022-35290-6)
Supplement: Supplementary file 1 — Supplementary Information [file 41467_2022_35290_MOESM1_ESM.pdf]

Supplementary information

Thermal-inert and ohmic-contact interface for high performance half-Heusler based thermoelectric generator

Ruiheng Liu[#123], Yunfei Xing[#12], Jincheng Liao[1], Xugui Xia[1], Chao Wang[1], Chenxi Zhu[1], Fangfang Xu[1], Zhi-Gang Chen[4], Lidong Chen[12], Jian Huang[*12], Shengqiang Bai[*12]

[1]State Key Laboratory of High Performance Ceramics and Superfine Microstructure, Shanghai Institute of Ceramics, Chinese Academy of Sciences, Shanghai, 200050, China.
[2]Center of Materials Science and Optoelectronics Engineering, University of Chinese Academy of Sciences, Beijing, 100049, China
[3]Shenzhen Institute of Advanced Electronic Materials, Shenzhen Institutes of Advanced Technology, Chinese Academy of Sciences, Shenzhen 518055, China
[4]School of Chemistry and Physics, Queensland University of Technology, Brisbane, Queensland 4001, Australia
# These authors contributed equally to this work.
*Correspondence and requests for materials should be addressed to J. H. (email: jhuang@mail.sic.ac.cn) or to S. B (email: bsq@mail.sic.ac.cn).

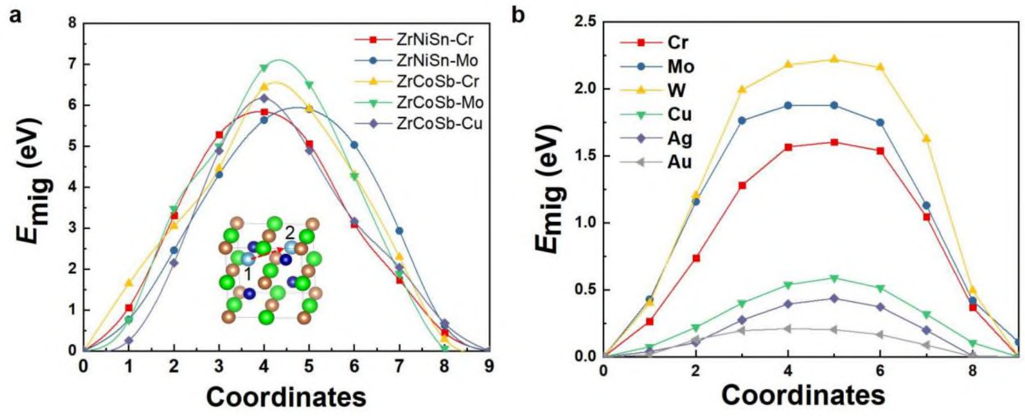

Supplementary Figure 1 The activation energy barrier (E_{Mig}). **a** Typical *d*-metal atoms from one tetrahedral vacancy site to neighboring unoccupied vacancy site in HHs. **b** Self-diffusion of selected *d*-metals via vacancy mechanism.

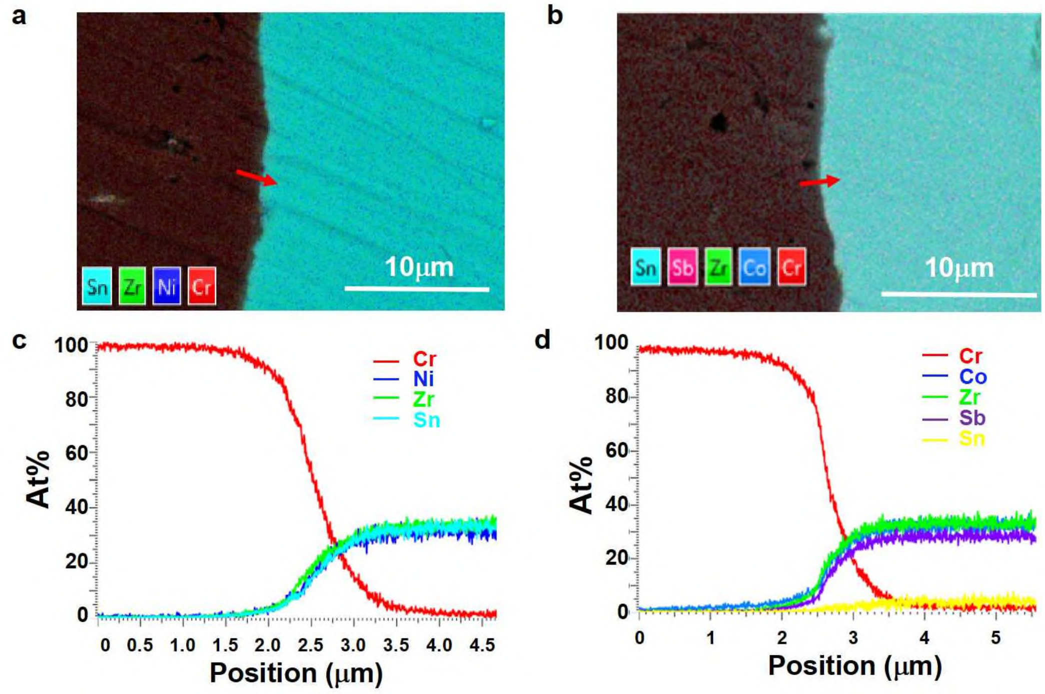

Supplementary Figure 2 Elemental mapping images and the compositional line profiles at interfaces. **a,c** Cr/$ZrNiSn_{0.99}Sb_{0.01}$ joints; **b,d** Cr/$ZrCoSb_{0.8}Sn_{0.2}$ joints. All the joints were aged at 1073K for 30 days.

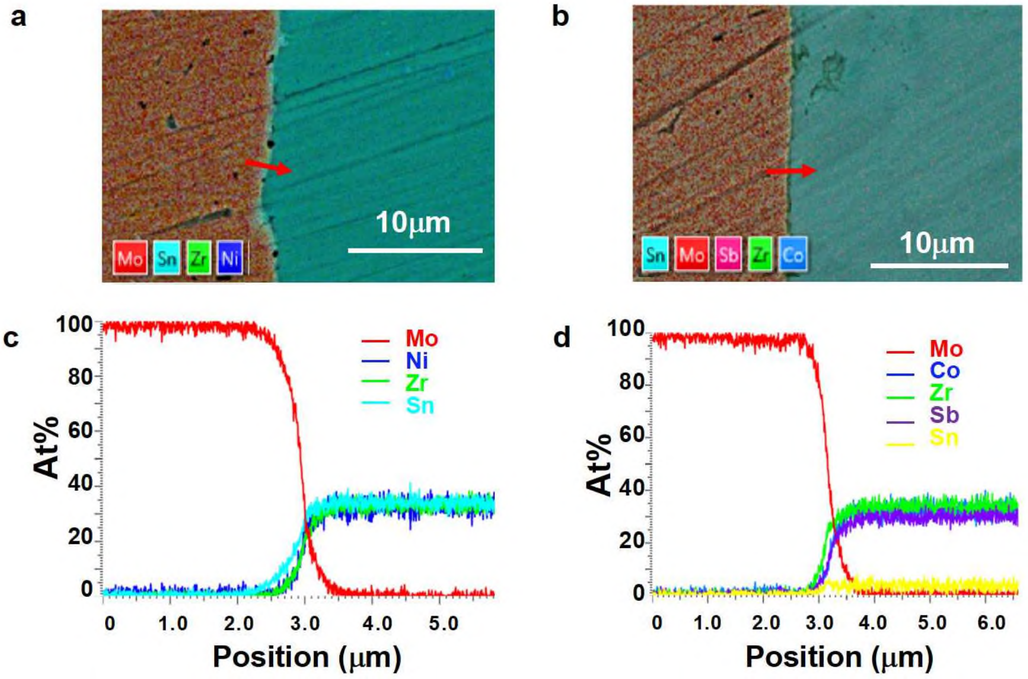

Supplementary Figure 3 Elemental mapping images and the compositional line profiles at interfaces. **a,c** Mo/$ZrNiSn_{0.99}Sb_{0.01}$ joints; **b,d** Mo/$ZrCoSb_{0.8}Sn_{0.2}$ joints. All the joints were aged at 1073K for 30 days.

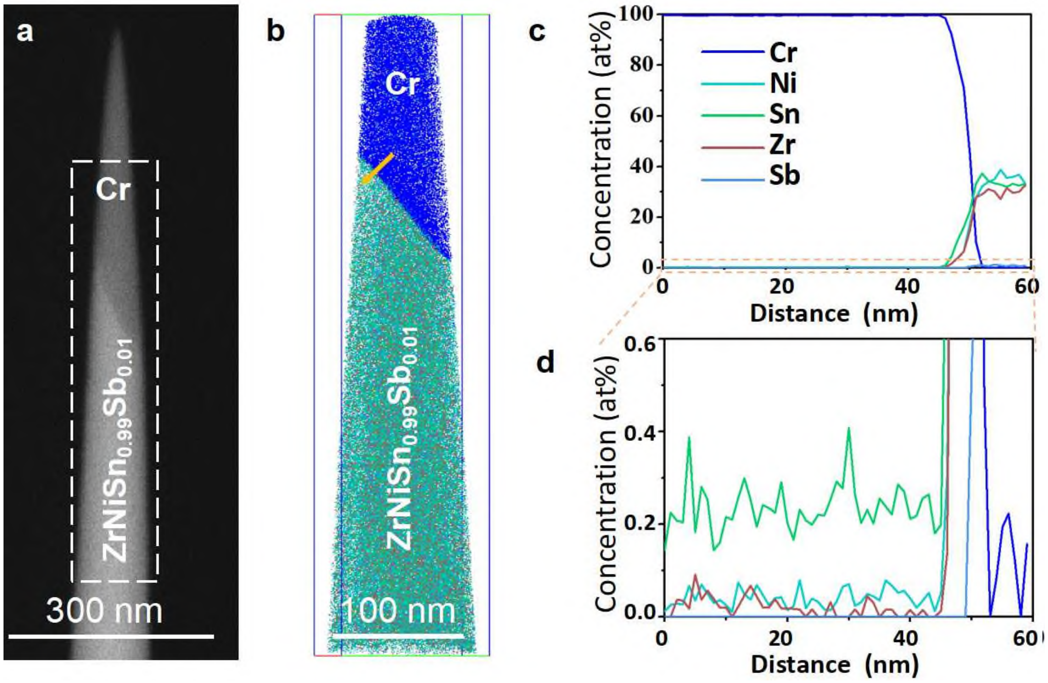

Supplementary Figure 4 3D-Atomic Probe Tomography detection for Cr/$ZrNiSn_{0.99}Sb_{0.01}$interface. **a** The image of APT tip samples fabricated by FIB milling; **b** 3D-atomic maps; **c,d** 1D concentration profiles at the interface.

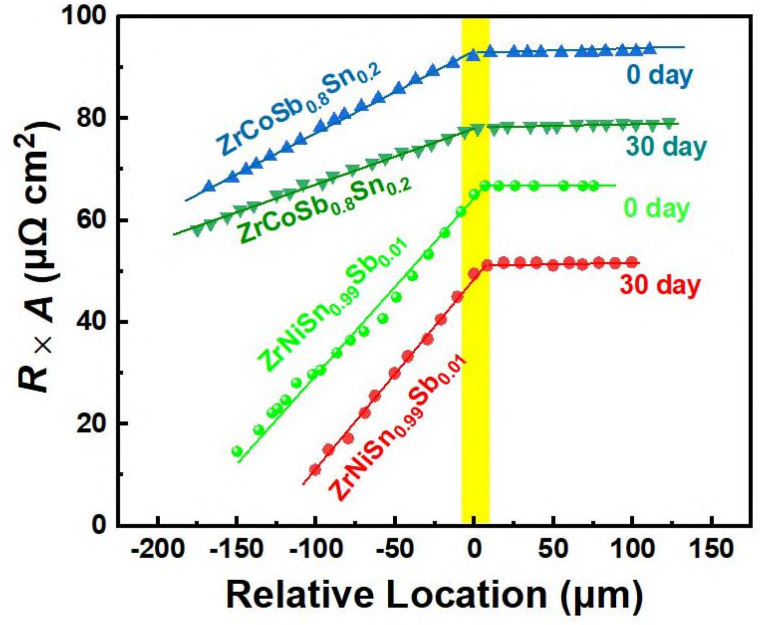

Supplementary Figure 5 The normalized resistance ($R \times A$, A is the cross section area of the joints) scans of $ZrNiSn_{0.99}Sb_{0.01}$/Mo and $ZrCoSb_{0.8}Sn_{0.2}$/Mo joints before and after aged at 1073 K for 30 days.

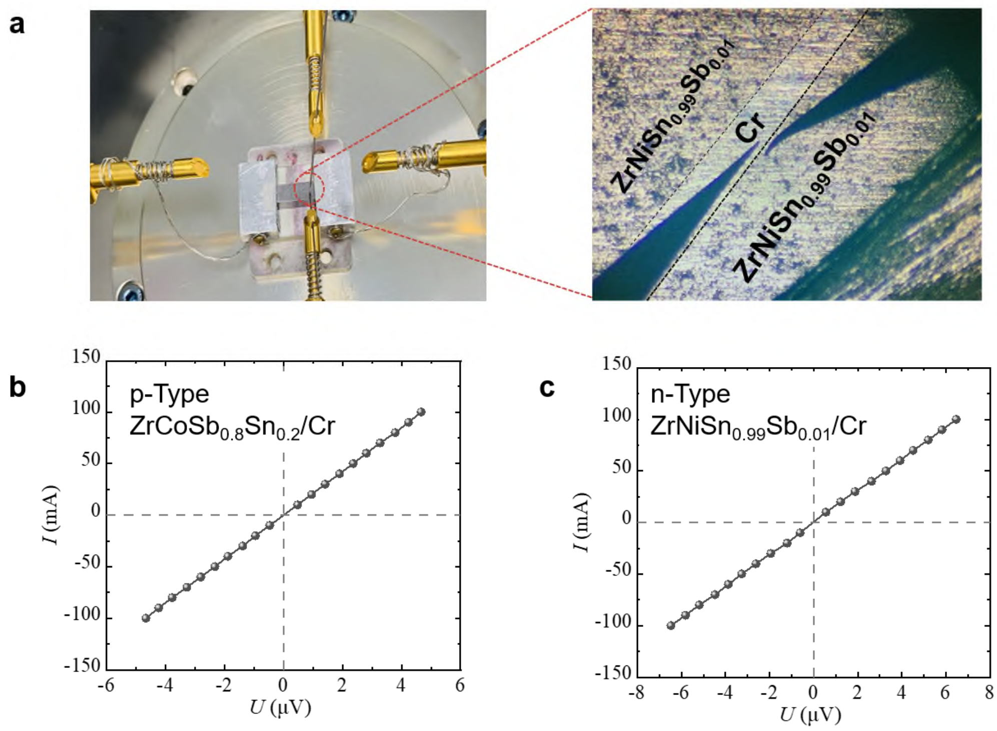

Supplementary Figure 6 I-V measurement of half-Heusler compounds and Cr interface. **a** The scene drawing of I-V measurement for p-type $ZrCoSb_{0.8}Sn_{0.2}$/Cr joint **b** I-V curve of p-type $ZrCoSb_{0.8}Sn_{0.2}$/Cr joint, and **c** I-V curve of n-type $ZrNiSn_{0.99}Sb_{0.01}$/Cr joint. The measurement was carried out at room temperature 300K.

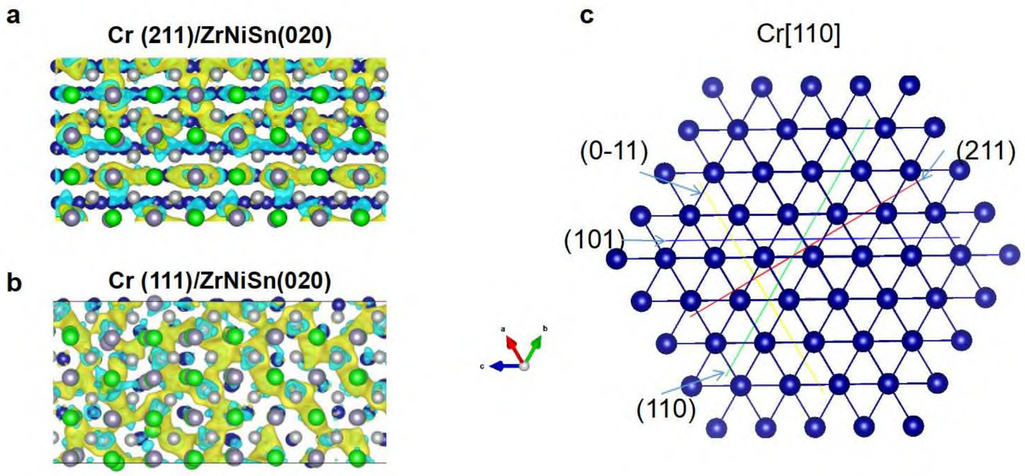

Supplementary Figure 7 Charge density difference. **a** Cr(211)/ZrNiSn(020); **b** Cr(111)/ZrNiSn(020) interfaces (top view), plot using a 0.005 e·$Å^{-3}$ isosurface; **c** The crystal orientation of Cr corresponding to the TEM investigation in Fig. 3c, showing the interface plane bonding with ZrNiSn(020) is the (211) plane.

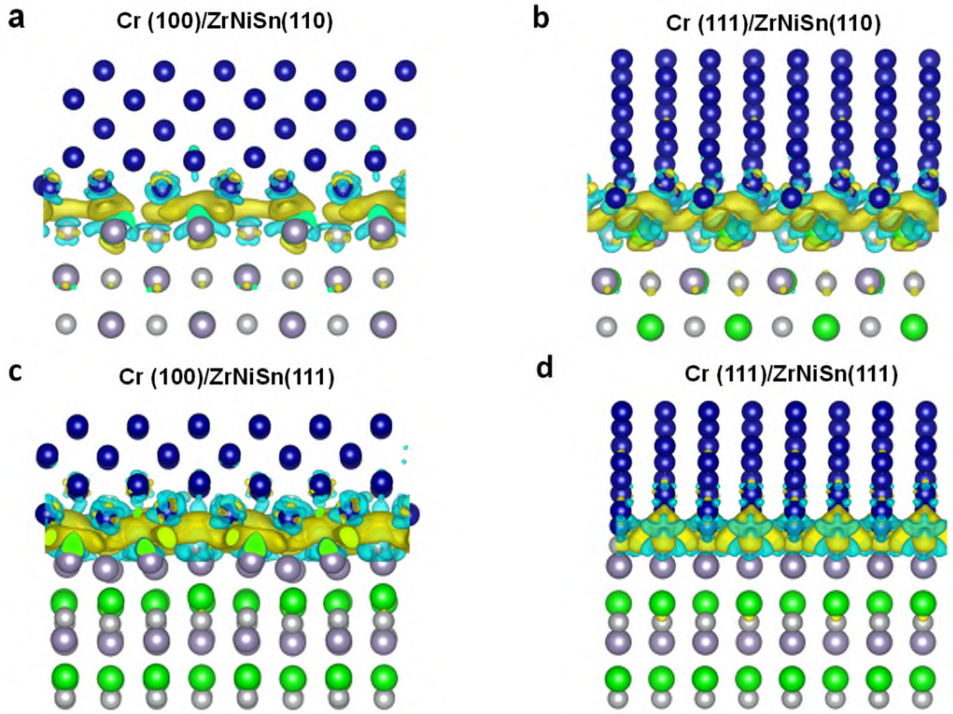

Supplementary Figure 8 Charge density difference of **a** Cr(100)/ZrNiSn(110), **b** Cr(111)/ZrNiSn(110), **c** Cr(100)/ZrNiSn(111), **d** Cr(111)/ZrNiSn(111) interfaces, plot using a 0.005 e·$Å^{-3}$ isosurface.

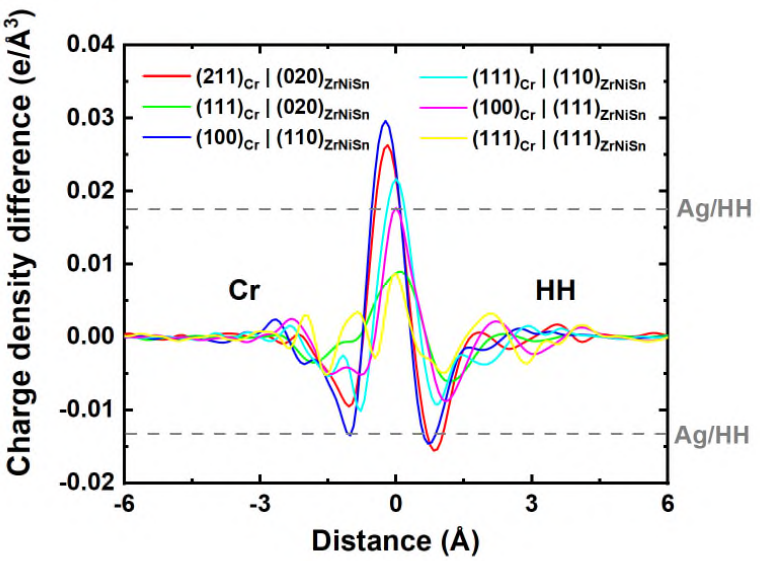

Supplementary Figure 9 Planar charge density difference as a function of z-axis distance of the interface for different boundary structures between ZrNiSn and Cr.

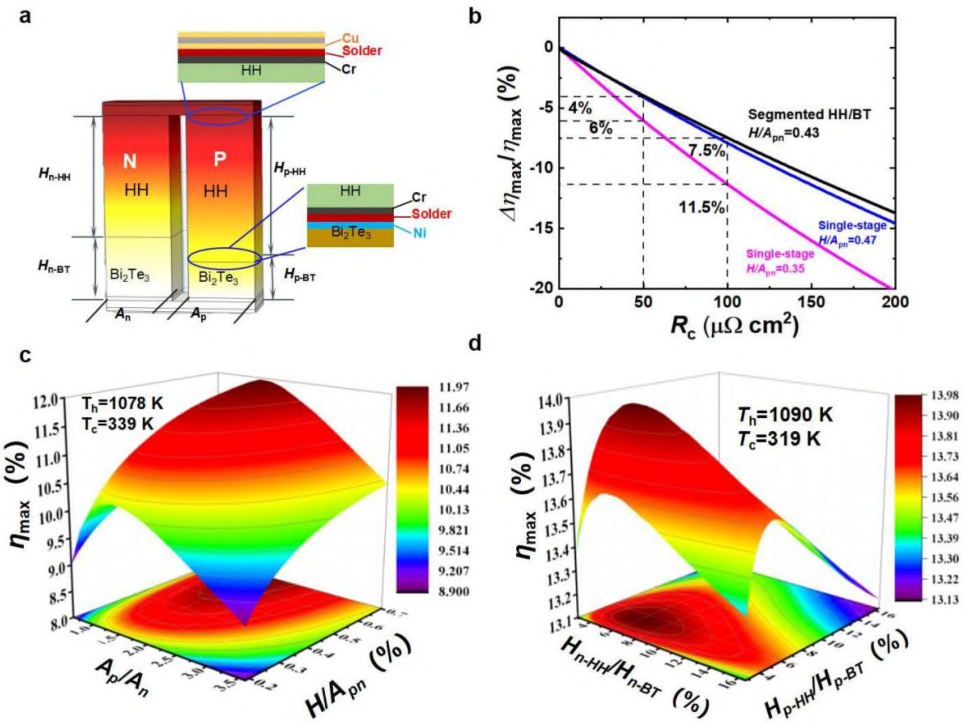

Supplementary Figure 10 Geometry optimization of TE module. **a** Schematic diagram of the module's topology and interface. **b** The loss of maximum conversion efficiency ($\Delta\eta_{max}/\eta_{max}$) as a function of H/A_{pn} and interfacial resistivity (R_c) for different module. **c** 3D plots of the calculated maximum conversion efficiency for HH-based single-stage module based on the boundary conditions specified by T_h=1078 K and T_c=339 K. **d** 3D plots of the calculated maximum conversion efficiency for HH/BT segmented module based on boundary conditions specified by T_h= 1090 K and T_c = 319 K.

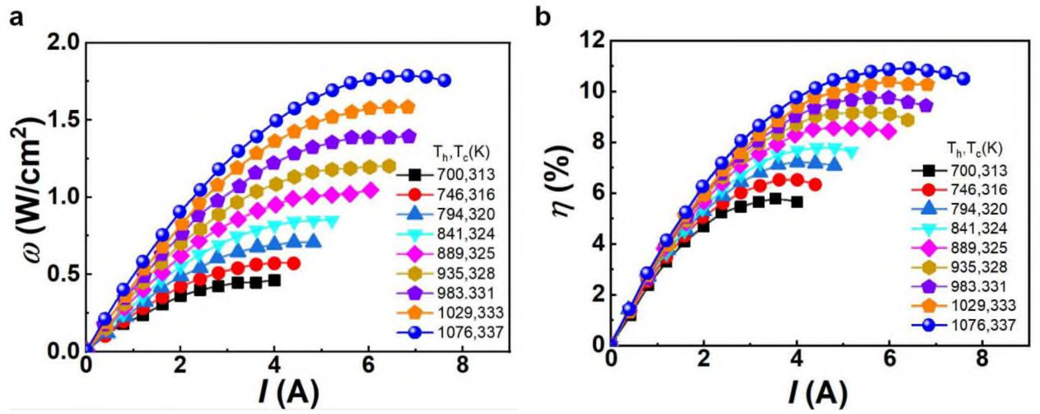

Supplementary Figure 11 Measured performance of the 8-pair single-stage module using Cr as barrier layer. **a** Output power density and **b** conversion efficiency as a function of current at different operating temperatures. T_h and T_c present the hot-side electrode temperature and the cold-side electrode temperature, respectively.

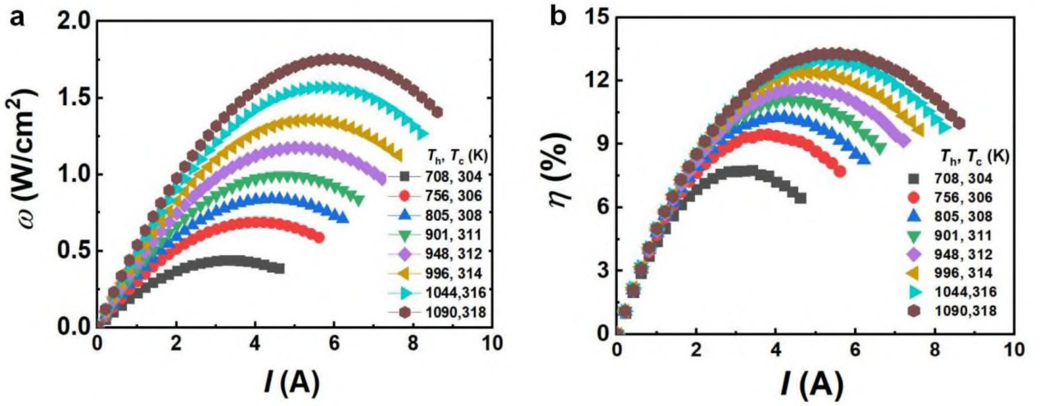

Supplementary Figure 12 Measured performance of the 8-pair HH/Bi_2Te_3 segmented module using Cr as barrier layer. **a** Output power density and **b** conversion efficiency as a function of current at different operating temperatures. T_h and T_c present the hot-side electrode temperature and the cold-side electrode temperature, respectively.

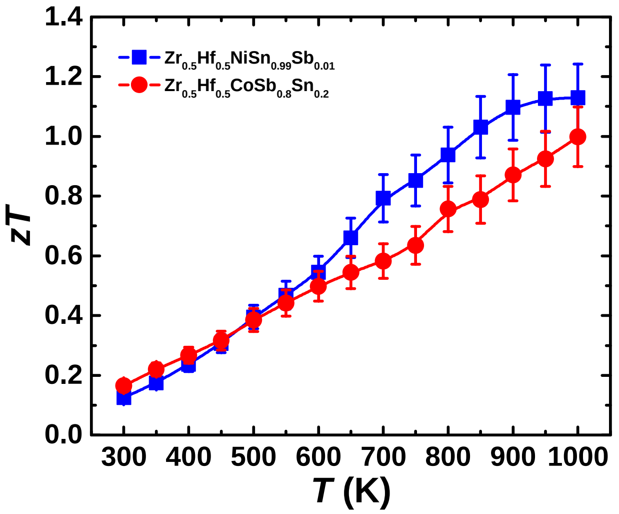

Supplementary Figure 13 The temperature dependent *ZT*s of p-type $Zr_{0.5}Hf_{0.5}CoSb_{0.8}Sn_{0.2}$ and n-type $Zr_{0.5}Hf_{0.5}NiSn_{0.99}Sb_{0.01}$.

Supplementary Table 1 The vacancy formation energy (E_{vac})of in ZrNiSn and ZrCoSb compounds.

ZrNiSn	E_{vac} (eV)	ZrCoSb	E_{vac} (eV)
Zr-vac	6.61	Zr-vac	5.65
Ni-vac	0.83	Co-vac	1.56
Sn-vac	2.07	Sb-vac	2.39

Supplementary Table 2 The substitution formation energy (E_{sub} in eV) of various transition metals in ZrNiSn and ZrCoSb compounds.

Compounds	Substitution site	Cr	Mo	W	Cu	Ag	Au
ZrNiSn	@Zr	3.96	3.12	3.50	4.04	3.95	3.92
	@Ni	4.01	4.81	5.43	1.26	2.00	0.88
	@Sn	4.50	4.37	4.51	3.17	3.07	2.17
	@vacancy	2.28	2.46	-	0.75	1.61	1.32
ZrCoSb	@Zr	4.00	3.51	3.82	3.99	4.10	4.04
	@Co	3.68	4.61	5.41	2.50	3.38	2.41
	@Sb	4.35	4.12	4.14	4.40	1.30	0.39
	@vacancy	2.73	3.02	3.97	1.70	2.82	2.74

Supplementary Table 3. The work functions of various transition metals.

Elements	Cr	Mo	W	Cu	Ag	Au
Work function (eV)	4.60	4.37	4.50	4.65	4.26	5.10

Supplementary Table 4. The summarization of the published module efficiency data, including of p-type and n-type materials, hot- and cold-side temperatures, electrode interlayer, conversion efficiency.

P-type leg	N-type leg	T_h (K)	T_c (K)	Inter -layer	η (%)
PbTe[3]	PbTe	873	303	$Co_{0.8}Fe_{0.2}$	~8
PbTe[3]	PbTe/Bi_2Te_3	873	283	$Co_{0.8}Fe_{0.2}$	~11
$Ge_{0.84}Pb_{0.1}Sb_{0.06}TeB_{0.07}$[4]	$Yb_{0.3}Co_4Sb_{12}$	708	283	Mo	5.64
$Ge_{0.84}Pb_{0.1}Sb_{0.06}TeB_{0.07}$[4]	$Yb_{0.3}Co_4Sb_{12}$	760	283	Mo	7.4
Ge0.96Bi0.04-Te)0.96(CuI)0.04[5]	$Bi_2Te_{2.7}Se_{0.3}$ + 0.097 wt%$BiCl_3$	700	300		8.6
FeNbSb[6]	ZrNiSn-based alloy	991	336		6.2
α-MgAgSb[8]	$Mg_3Sb_{1.5}Bi_{0.5}$	593		Cu	7.3
$Nb_{0.86}Hf_{0.14}FeSb$[12]	$Zr_{0.5}Hf_{0.5}NiSn_{0.97}Sb_{0.03}$	1046	366		10.5
$Hf_{0.3}Zr_{0.7}CoSn_{0.3}Sb_{0.7}$/nano-$ZrO_2$[25]	$Hf_{0.6}Zr_{0.4}NiSn_{0.995}Sb_{0.005}$	1021	317		8.1
$Hf_{0.3}Zr_{0.7}CoSn_{0.3}Sb_{0.7}$/nano-$ZrO_2$[25]	$Hf_{0.6}Zr_{0.4}NiSn_{0.995}Sb_{0.005}$	970	313		8.7
$Zr_{0.5}Hf_{0.5}CoSb_{0.8}Sn_{0.2}$ [26]	$Hf_{0.6}Zr_{0.4}NiSn_{0.995}Sb_{0.005}$	800	300		5
MCoSn[27]	MNiSn	743	320		4
$Zr_{0.5}Hf_{0.5}CoSb_{0.8}Sn_{0.2}$[28]	$Zr_{0.5}Hf_{0.5}NiSn_{0.985}Sb_{0.015}$	953			8.3
$Zr_{0.5}Hf_{0.5}CoSb_{0.8}Sn_{0.2}$+$Bi_2Te_3$[28]	$Zr_{0.5}Hf_{0.5}NiSn_{0.985}Sb_{0.015}$+$Bi_2Te_3$	1013	315		12.4
Ce0.85Fe3CoSb12/1.4 vol% rGO[30]	$Yb_{0.27}Co_4Sb_{12}$/0.72 vol% rGO	873	296		8.4
$(Mn_{0.98}$-$Mo_{0.02})(Si_{0.9865}Al_{0.0035}Ge)_{1.74}$[29]	$Mg_2Si_{0.4}Sn_{0.6}$	823	303		7.3
La0.7Ba0.1Ga0.1Ti0.1Fe3Co1Sb12[31]	$Yb_{0.3}Ca_{0.1}Al_{0.1}Ga_{0.1}In_{0.1}Co_{3.75}Fe_{0.25}Sb_{12}$	873	323		8
(AgSbTe2)0.15(GeTe)0.85[32]	PbTe	773	363		6
$Bi_{0.4}Sb_{1.6}Te_3$/$CeFe_{3.85}Mn_{0.15}Sb_{12}$[33]	$Bi_2Te_{2.5}Se_{0.5}$/$Yb_{0.3}Co_4Sb_{12}$	849	308		12
$Bi_xSb_{2-x}Te_3$/$Ag_{0.9}Pb_9Sn_9Sb_{0.6}Te_{20}$[34]	$Bi_2Te_{3-x}Se_X$/$Ag_{0.86}Pb_{19+x}SbTe_{20}$	670	312		~6.5
					6
$[(AgSbTe_2)_{(1-x)}(GeTe)_x]$/PbTe[35]	Bi_2Te_3	773	293		>10
$(Ge_{0.98}Cu_{0.04}Te)_{0.88}(PbSe)_{0.12}$[7]	$Mg_{3.05}Y_{0.015}SbBi$	600	280	SnTe	~10
Be_2Te_3[36]	$Hf_{0.75}Zr_{0.25}NiSn_{0.99}Sb_{0.01}$	875	293		12
